# A Novel Expression Signature from the Perspective of Mesenchymal-Epithelial Transition for Hepatocellular Carcinoma with Regard to Prognosis, Clinicopathological Features, Immune Cell Infiltration, Chemotherapeutic Efficacy, and Immunosuppressive Molecules

**DOI:** 10.1155/2021/5033416

**Published:** 2021-07-28

**Authors:** Lijun Xu, Qing Zheng

**Affiliations:** Key Laboratory of Gastroenterology and Hepatology, Ministry of Health, Inflammatory Bowel Disease Research Center, Shanghai Institute of Digestive Disease, Division of Gastroenterology and Hepatology, Renji Hospital, School of Medicine Shanghai Jiao Tong University, 160# Pu Jian Ave, Shanghai 200127, China

## Abstract

**Purpose:**

Mesenchymal-epithelial transition (MET), a reverse biological process to epithelial-mesenchymal transition (EMT), is involved in tumor metastasis and invasion. However, the role of MET-related genes (MRGs) in hepatocellular carcinoma (HCC) prognosis remains unclear.

**Methods:**

In this research, we obtained MRGs data and clinical information from public databases. In the TCGA dataset, a prognostic signature for HCC was constructed by the least absolute shrinkage and selection operator (LASSO) method and externally verified using the ICGC dataset.

**Results:**

There were 148 differentially expressed MRGs (DEMRGs), out of which 37 MRGs were found associated with overall survival (OS) in the univariate Cox analysis. A novel signature integrating of 5 MRGs was constructed, which split patients into high- and low-risk groups. Kaplan–Meier analysis revealed that high-risk patients had unfavorable OS than those low-risk counterparts. Receiver operating characteristic curve (ROC) showed great performance of this signature in predictive ability. Multivariate Cox analysis confirmed that this signature could independently predict HCC prognosis. The analysis of immune cell infiltration demonstrated that immune status varied differently between high- and low-risk groups. The analysis of clinicopathological characteristics suggested that tumor grade, clinical stage, and T stage were different between risk groups. The analysis between this signature and chemotherapeutic efficacy and immunosuppressive molecules indicated that this signature could serve as a promising predictor.

**Conclusions:**

In conclusion, we constructed and verified a novel signature from the perspective of MET, which was significantly associated with HCC prognosis, clinicopathological features, immune status, chemotherapeutic efficacy, and immunosuppressive biomarkers.

## 1. Introduction

Hepatocellular carcinoma (HCC) is the most pervasive type of primary liver cancer [1], and its incidence and fatality rate are the fourth and second among all types of malignant tumors [2, 3]. Currently, many well-known risk factors could contribute to HCC development, such as chronic infection with hepatitis B and C viruses, nonalcoholic steatohepatitis, alcohol intake, and ingestion of fungal toxins such as aflatoxin B1. Although a tremendous progress has been made in medical, locoregional, and surgical therapies, unfavorable prognosis is still a serious problem for HCC [4]. Besides, the recurrence and metastasis of tumor and drug resistance lead to an unfavorable 5-year overall survival (OS) rate. Thus, it is urgently required to identify novel prognostic predictors for HCC.

Mesenchymal-epithelial transition (MET) refers to a biological process in which the epithelial-mesenchymal transition (EMT) cells revert to epithelial phenotype to successfully colonize an organ and cause secondary lesions [5]. During the MET process, EMT cells lose their motile properties, adopt an apicobasal polarization, and reexpress the junctional complexes [6]. It has been established that the sequential EMT-MET process is required for cancer cells to relocate into the distant metastasis site, resulting in poor survival outcome [7]. Thus, both EMT- and MET-related genes could serve as promising predictors for patients' prognosis. Chen and Zhao constructed and verified a prognostic expression signature for HCC from the perspective of EMT [8]. However, the role of MET-related genes (MRGs) in HCC remains unclear.

Thus, our study focused on the development of a prognostic expression signature from the perspective of MET. First, both expression profiles of MRGs and clinical data of HCC samples were obtained from TCGA and ICGC datasets. Then, a novel expression signature was constructed using prognostic differentially expressed MRGs (DEMRGs) obtained from the TCGA dataset and externally validated using the ICGC dataset. Finally, the enrichment analysis of immune cell features and immune function characteristics was performed to better understand the role of tumor immunity in this signature.

## 2. Materials and Methods

### 2.1. Data Acquisition

MRGs were obtained from GeneCards website (https://www.genecards.org/) [9]. RNA sequencing data and clinical data for HCC were downloaded from the TCGA-LIHC project (https://portal.gdc.cancer.gov/repository) and ICGC database (https://dcc.icgc.org/releases/current/Projects/LINC-JP). Expression profiles of MRGs extracted from TCGA and ICGC datasets were used for further analysis.

### 2.2. Identification of Prognostic MRGs

DEMRGs between HCC samples and normal ones were recognized using “limma” R package [10] in the TCGA dataset. We set false discovery rate (FDR) <0.05 and |log2 fold change (FC)| >1 as the threshold. Univariate Cox regression analysis was then performed to screen out the MRGs correlated with OS, and *P* < 0.01 was regarded as the statistical difference. Venn diagram was plotted in which the interaction between DEMRGs and MRGs with the prognostic value was displayed and used for subsequent analysis. Correlation analysis among these prognosis-related DEMRGs was performed, and the protein-protein interaction (PPI) network was analyzed in the STRING database (https://www.string-db.org/) [11] to identify the hub genes. We set medium confidence (0.400) as the minimum required interaction score.

### 2.3. Construction of MRGs-Related Signature and Evaluation of Its Clinical Utility

The least shrinkage and selection operator (LASSO) method with tenfold cross-validation was performed to reduce the risk of overfitting and select the optimal predictors for OS of TCGA-LIHC project [12, 13]. Some MRGs with a regression coefficient of nonzero were incorporated into this novel signature, whose risk score was calculated based on MRGs expression value multiplied by their corresponding regression coefficient. The patients were then split into 2 groups (high- or low-risk) with a cutoff point of the median risk score. The Wilcoxon signed-rank test was conducted to analyze the difference of clinicopathological characteristics between high- and low-risk groups. The chi-square test was conducted to investigate the relationship between clinicopathological features and the risk score.

### 2.4. Investigation of the Role of This Novel Signature in Chemotherapeutic Efficacy and Immunosuppressive Molecules

The half inhibitory centration (IC_50_) of common antitumor drugs, such as doxorubicin, mitomycin C, sorafenib, cisplatin, and vinblastine, were calculated, and the Wilcoxon signed-rank test was conducted to explore the drug sensitivity between different risk groups. To visualize the relationship between this signature and the expression value of immune checkpoint inhibitors (ICIs)-related molecules, we applied “ggpubr” package to transform results into the violin plot.

### 2.5. Verification of This Novel Signature

Kaplan–Meier analysis was conducted to analyze the difference in OS between different risk groups. The distribution of risk score and survival outcome for each HCC patient was visualized using R software. Area under the curve (AUC) of 1-, 2-, and 3-year receiver operating characteristic curve (ROC) was calculated to assess the predictive ability of this novel signature using “timeROC” package. Principal component analyse (PCA) and t-SNE analysis were performed to investigate whether MRGs identified by this signature could distinguish HCC samples between different risk groups. Univariate and multivariate Cox analyses were performed to confirm whether this signature could predict prognosis independent of clinicopathological indicators.

### 2.6. Functional Enrichment Analysis and Tumor-Infiltrating Immune Cells

R “limma” package was used to determine DEMRGs between different risk groups. We selected FDR <0.05 and |log2 FC| >1 as the threshold. Gene ontology (GO) and Kyoto Encyclopedia of Genes and Genomes (KEGG) analyses were then performed to understand the biological function and pathways. To investigate the immune infiltration landscape between different risk groups, single-sample gene set enrichment analysis (ssGSEA) was implemented to calculate the score of 16 infiltrating immune cells and 13 immune functions [14].

## 3. Results

### 3.1. Identification of Prognosis-Related MRGs

**Figure 1:**
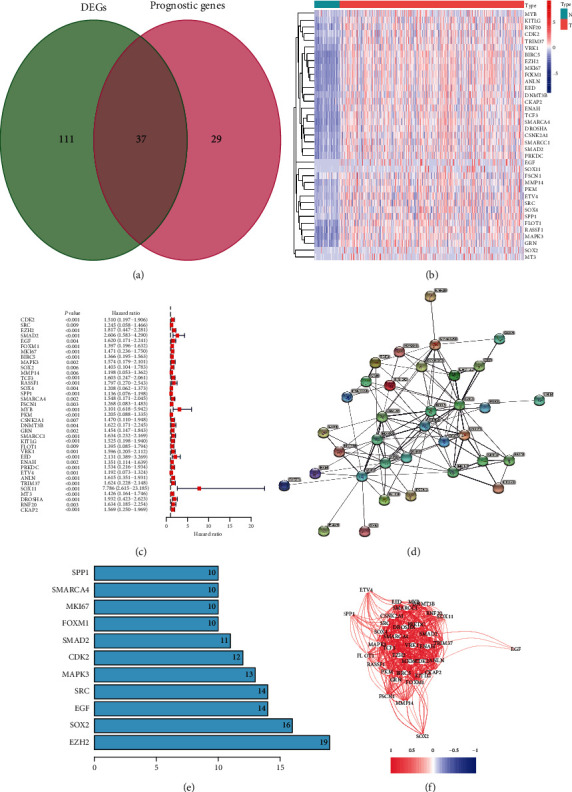
Identification of prognosis-related DEMRGs in the TCGA-LIHC dataset. (a) The Venn diagram presenting DEMRGs which were associated with OS in the univariate Cox regression analysis. (b) The heatmap showing 37 prognosis-related DEMRGs. (c) The forest plot displaying the relationship between 37 prognosis-related DEMRGs and OS in the univariate Cox regression analysis. (d) The PPI network among candidate genes obtained from the STRING database. (e) Hub genes with the top 11 degrees of interaction. (f) The correlation analysis of candidate genes.

A total of 365 and 231 HCC samples with the available gene expression value and clinical information were retrieved from TCGA and ICGC dataset, respectively. The detailed clinical characteristics of these samples are summarized in Table 1. 148 DEMRGs between 374 HCC tissues and normal ones were identified, and 37 out of them were found associated with OS in the univariate Cox analysis (Figures 1(a)–1(c)). The interactions among 37 prognostic MRGs were visualized in the PPI network, in which there were 37 nodes and 111 edges (Figure 1(d)). Genes with the top 11 degrees of interaction were identified as hub genes (Figure 1(e)). The correlation among 37 prognostic MRGs is shown in Figure 1(f).

### 3.2. Construction of MRGs-Related Signature

Based on the expression profiles of 37 prognosis-related MRGs, we performed LASSO regression analysis to construct a prognostic expression signature, in which a total of 5 MRGs, whose coefficients were non-zero, were regarded as optimal predictors. The formula for this novel signature is given as follows: [EZH2 expression*∗* (0.231780268980573)] + [SPP1 expression *∗* (0.0622786294410273)] + [ETV4 expression *∗* (0.0203905514170323)] + [ANLN expression *∗* (0.124600566473429)] + [MT3 expression *∗* (0.169607630930886)]. The median risk score was then used as a cutoff value to split HCC samples into 2 groups (high- and low-risk). A total of 182 high- and 183 low-risk cases in the TCGA dataset were identified for subsequent analysis.

### 3.3. Clinical Utility of This Novel Signature

**Figure 2:**
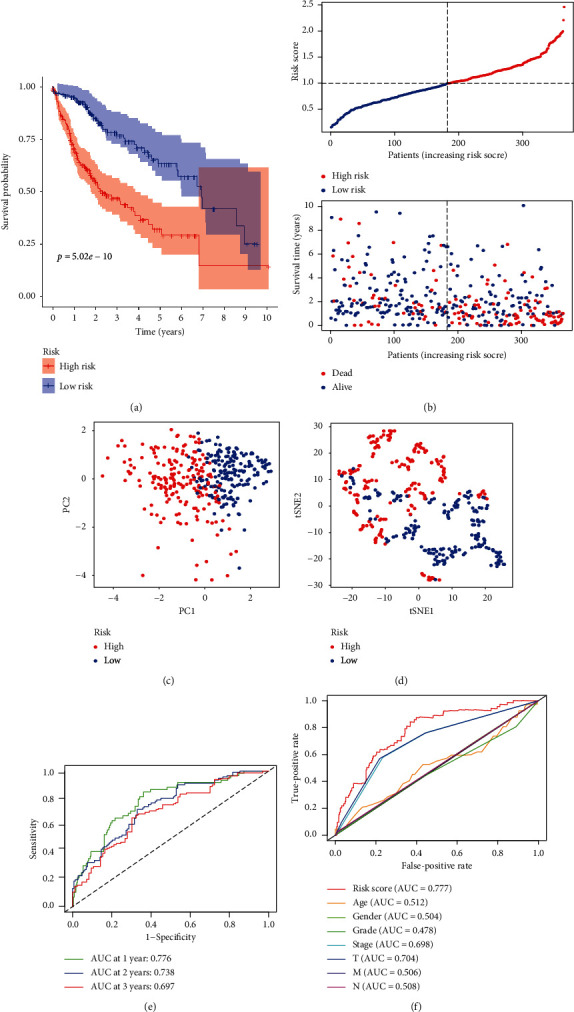
Development of a MRGs expression signature in the TCGA dataset. (a) The Kaplan–Meier curve survival analysis. (b) The risk score curve plot and risk score scatter plot of high- and low-risk HCC patients. (c) PCA plot of the TCGA dataset. (d) t-SNE analysis of the TCGA dataset. (e) AUC of time-dependent ROC used to assess performance of this signature in predictive ability. (f) AUC of this signature and clinicopathological parameters.

**Figure 3:**
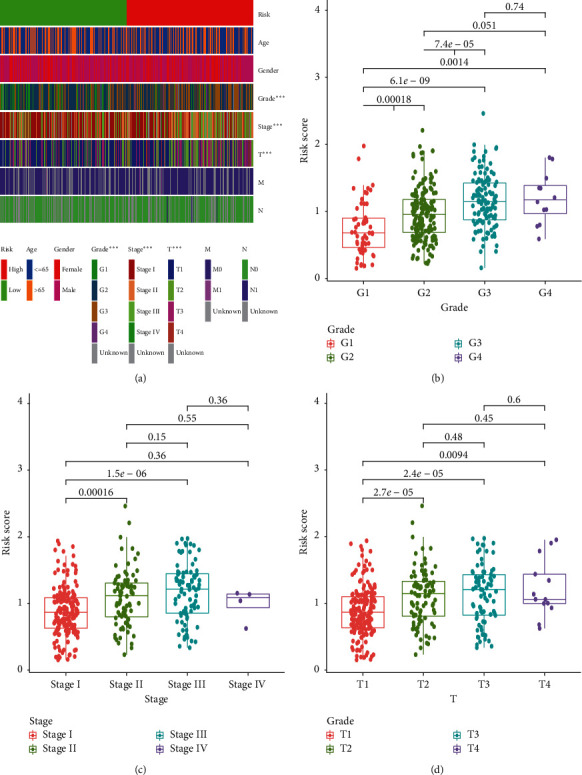
Clinicopathological features of this novel expression signature. Clinical characteristics (a), including tumor grade (b), clinical stage (c), and T stage (d) significantly associated with the risk and risk score.

Kaplan–Meier analysis demonstrated that high-risk patients had shorter OS than those low-risk counterparts (Figure 2(a)). Patients with low-risk were less likely to suffer from earlier death compared with those high-risk counterparts (Figure 2(b)). PCA and t-SNA analysis revealed that it was easy to distinguish HCC samples between high- and low-risk group (Figures 2(c) and 2(d)). The AUC of 1-, 2-, and 3-year ROC for this novel signature was 0.776, 0.738, and 0.697 (Figure 2(e)). By comparison with ROC curve of this signature and other clinicopathological parameters, we found that the AUC of this signature was higher than that of clinical indicators (Figure 2(f)). Besides, the chi-square test and Wilcoxon signed-rank test revealed that clinicopathological characteristics, including tumor grade, clinical stage, and T stage were different between high- and low-risk groups (Figure 3).

### 3.4. Estimation of This Novel Signature and the Efficacy of Chemotherapeutics and the Expression Value of Immunosuppressive Molecules

**Figure 4:**
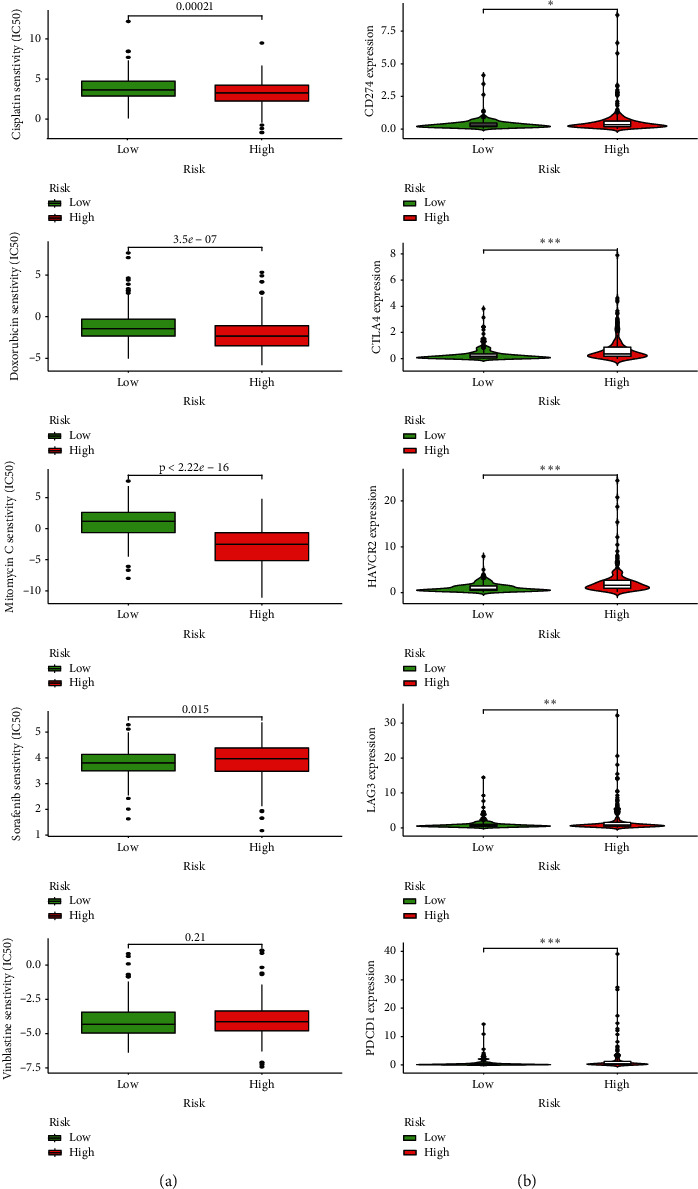
The relationship between this signature and chemotherapeutic efficacy and ICIs-related molecules. (a) Low-risk group having a higher IC50 of cisplatin, doxorubicin, and mitomycin and a lower IC50 of sorafenib. (b) High-risk group positively related with PDCD1, CD274, CTLA4, HAVCR2, and LAG3.

By exploring the relationship between this signature and the efficacy of common chemotherapeutics used for HCC, we found that the low-risk group had a higher IC50 of cisplatin, doxorubicin, and mitomycin and a lower IC50 of sorafenib (Figure 4(a)). Because, ICIs were the common treatment for HCC in clinical practice, we investigated whether this signature was associated with ICIs-related molecules and found that the high-risk group was positively related with PDCD1, CD274, CTLA4, HAVCR2, and LAG3 (Figure 4(b)).

### 3.5. External Verification of This Novel Signature

**Figure 5:**
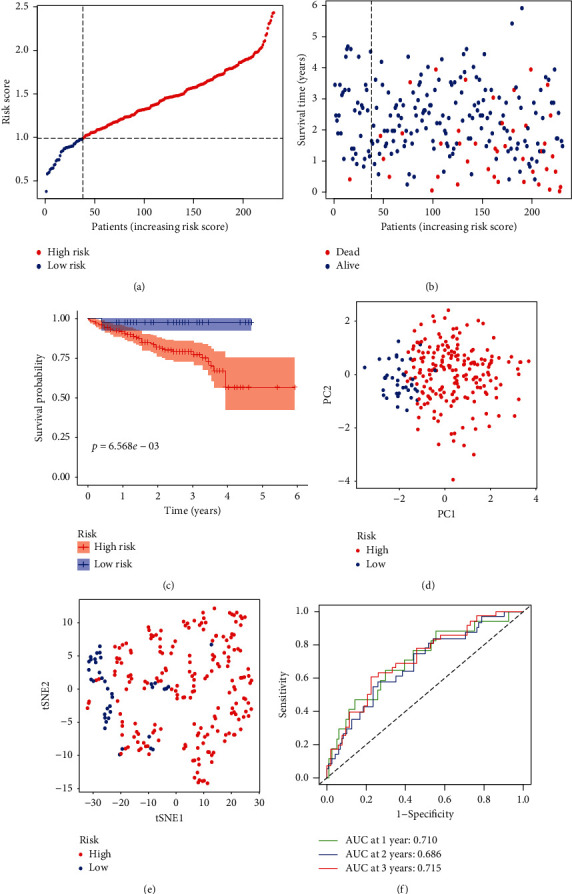
Validation of this novel expression signature in the ICGC dataset. (a) The risk score curve plot in the ICGC dataset. (b) The risk score scatter plot of high- and low-risk HCC patients. (c) The Kaplan–Meier curve survival analysis. (d) PCA plot of the ICGC dataset. (e) t-SNE analysis of the ICGC dataset. (f) AUC of time-dependent ROC used to assess performance of this signature in predictive ability in the ICGC dataset.

Subsequently, the ICGC dataset was used as external validation to assess the predictive ability of this novel signature. By calculating the risk score of each HCC sample based on the formula for this signature, we split them into 2 groups (high- or low-risk) with a cutoff of the median value (Figure 5(a)). Patients with low risk had a lower probability of earlier death and favorable OS than those high-risk counterparts (Figures 5(b) and 5(c)), which were in great accord with the results of the TCGA dataset. Likewise, PCA and t-SNE analysis revealed that HCC samples in different risk groups were easily distinguished (Figures 5(d) and 5(e)). Besides, the AUC of 1-, 2-, and 3-year ROC for this signature was 0.710, 0.686, and 0.715 (Figure 5(f)).

### 3.6. Independent Prognostic Analysis of This Signature

**Figure 6:**
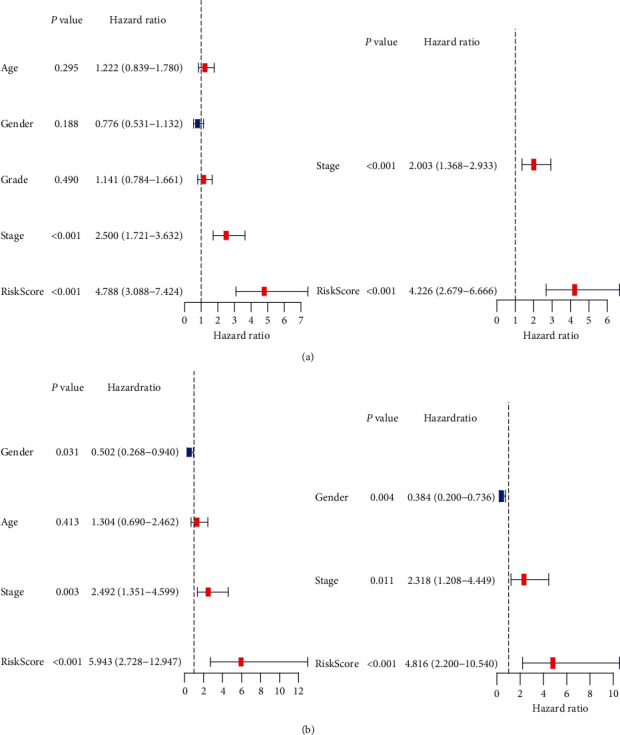
Results of univariate and multivariate Cox regression analysis of OS in the TCGA development dataset (a) and the ICGC validation dataset (b).

To investigate whether this signature could predict HCC prognosis independent of clinicopathological parameters, univariate and multivariate Cox analyses were performed. The results revealed that this signature was remarkedly associated with OS (TCGA dataset: HR = 4.788, 95% CI = 3.088–7.424, *p* < 0.001; ICGC dataset: HR = 5.943, 95% CI = 2.728–12.947, *p* < 0.001) and could serve as an independent predictor for OS in both TCGA and ICGC datasets (HR = 4.226, 95% CI = 2.679–6.666, *p* < 0.001; HR = 4.816, 95% CI = 2.200–10.540, *p* < 0.001, respectively) (Figure 6).

### 3.7. Functional Enrichment Analysis

**Figure 7:**
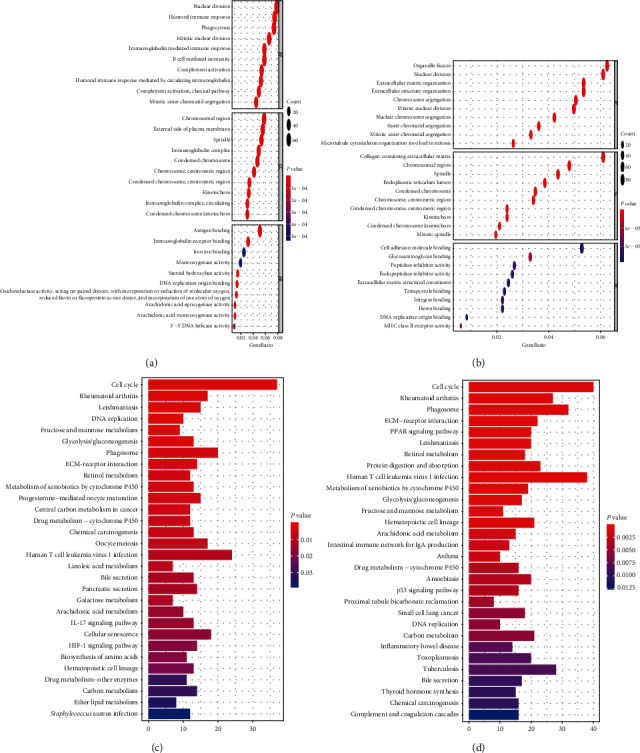
Representative results of GO and KEGG analyses. The most significant GO enrichment and KEGG pathways in the TCGA dataset (a, c) and ICGC dataset (b, d) are displayed.

To analyze the biological significance associated with this signature, we performed GO and KEGG analyses of DEMRGs between different risk groups. In both TCGA and ICGC datasets, the GO enrichment terms for the biological process were nuclear division, mitotic sister chromatid segregation, and mitotic nuclear division; for cellular component were chromosomal region, condensed chromosome, centromeric region, and kinetochore; and for molecular function was DNA replication origin binding (Figures 7(a) and 7(b)). Besides, the KEGG results showed that these DEMRGs of both datasets were mainly involved in pathways associated with cell cycle and DNA replication (Figures 7(c) and 7(d)).

### 3.8. Immune Cell Infiltration

**Figure 8:**
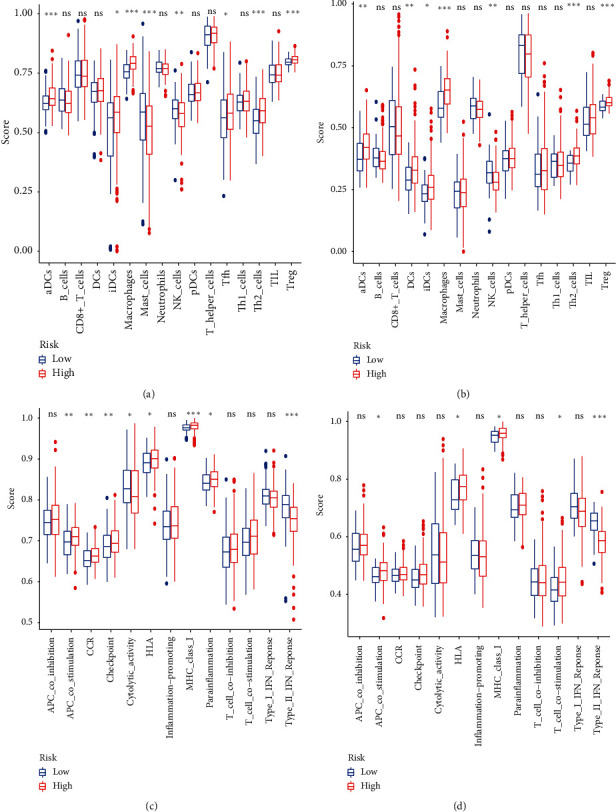
Comparison of the ssGSEA scores between different risk groups in the TCGA dataset and ICGC dataset. The scores of 16 immune cells (a, b) and 13 immune-related functions (c, d) are displayed in boxplots. Adjusted *p* values are shown. ns, not significant. ^*∗*^*P* < 0.05, ^*∗∗*^*P* < 0.01, ^*∗∗∗*^*P* < 0.001.

To elucidate the relationship between this signature and immune status, we implemented ssGSEA to calculate the risk score of immune cells and immune functions. Macrophages, aDCs, iDCs, Th2_cells, and Treg earned high scores in high-risk patients, while the score of NK_cells was higher in low-risk patients of TCGA and ICGC datasets. Noticeably, tumor-associated neutrophils, an essential component of tumor microenvironment contributing to immunosuppression, exhibited no significant difference between high- and low-risk groups (Figures 8(a) and 8(b)). Besides, the score of APC_co_stimulation, HLA, and MHC_class_I was higher in high-risk patients, while Type_II_IFN_Response earned a higher score in low-risk patients of TCGA and ICGC datasets (Figures 8(c) and 8(d)).

## 4. Discussion

The sequential EMT-MET process is essential for tumor cell metastasis, poor survival outcome, and drug resistance [15]. Numerous studies have focused on the development of prognostic expression signature from the perspective of EMT [8, 16]. However, fewer studies addressed the prognostic value of MRGs in cancer, especially for HCC.

In this study, bioinformatic analysis was conducted to investigate the role of 415 MRGs in HCC and their relationship with prognosis. A total of 148 DEMRGs were identified between HCC tissues and normal ones, and 25% (37/148) was found associated with OS in the univariate Cox analysis, suggesting that MET played a critical role in HCC. A novel gene expression signature integrating of 5 MRGs was constructed and verified using an external cohort with regard to its predictive ability for prognosis and relationship with clinicopathological characteristics. The analysis of immune cell infiltration revealed that this signature was significantly associated with tumor immunity. Besides, this signature was correlated with sensitivity of common antitumor drugs, including cisplatin, doxorubicin, mitomycin, and sorafenib and the expression value of immunosuppressive molecules, including PDCD1, CD274, CTLA4, HAVCR2, and LAG3, indicating that it could serve as a potential predictor for drug efficacy and ICIs-related biomarkers.

A total of 5 MRGs were finally incorporated into this novel signature, some of which have been reported to play an essential role in HCC tumorigenesis. For instance, EZH2, a family member of polycomb group protein, could regulate DNA and histone methylation to modify transcription epigenetically [17, 18]. In HCC, EZH2 could repress miR-22 at the epigenetic level to facilitate galectin-9 upregulation, resulting in tumorigenesis and aggressiveness [19]. SPP1 belongs to a secreted phosphoprotein, which possesses cell-adhesive and chemotactic properties [20]. In HCC, SPP1 is regarded as an essential regulator participating in enhancing HCC cell growth [21]. ETV4, a member of ETS family, could bind to the promoter region of downstream target genes to promote their transcription [22–24]. In HCC, PBK could promote invasion and metastasis by enhancing the binding of ETV4 to the uPAR promoter to activate its transcription [25]. ANLN is a conserved protein which could bind to cytoskeletal components and their regulator [26]. In HCC, miR-15a and miR-16-1 could bind to complementary sites in the 3'-UTRs of ANLN to inhibit tumor growth and predict favorable survival outcome [27].

To investigate the relationship between this novel signature and immune cell infiltration, we performed ssGSEA based on DEMRGs between different risk groups. The result revealed that the high-risk group of both TCGA and ICGC datasets had higher contents of macrophages, Th2_cells, and Treg. It is widely accepted that macrophages [28, 29], Th2_cells [30], and Treg [28, 31] could promote tumor propagation and invasion and are associated with unfavorable prognosis. Especially, CD4+ CD25+ Foxp3+ regulatory T cells could secrete inhibitory cytokines (IL-10 and TGF-*β*) to suppress NK cells and CD8+ T cells activity, resulting in T cell exhaustion and the defective antitumor effect [32]. Moreover, Type_II_IFN_Response and NK_cells were higher in low-risk patients. It is possibly because type II IFN is mainly released by NK_cells [33], which are major components of innate and adaptive immune defense against tumorigenesis [34]. Besides, the antigen-presenting process, including APC_co_stimulation, iDCs, aDCs, MHC_Class_I, and HLA, earned a high score in the high-risk group. One speculation is that mesenchymal cells have to revert to epithelial status to form metastatic colonization. This process is required by reactivation of signaling pathway and attachment between heterologous cells and healthy tissue, resulting in activation of antigen-presenting cells and promotion of antitumor T cell activity [6].

Several limitations should be recognized in our research. First, we constructed and verified a novel MRGs signature using TCGA and ICGC dataset. Real-world cohort is required to assess its accuracy and efficacy in future. Second, this novel expression signature is constructed merely from the hallmark of MET, and many genes with the prognostic value in HCC may be neglected. Finally, the potential mechanisms between MRGs identified by this signature and immune activity are not elucidated, and future experimental studies are needed to address this problem.

## 5. Conclusions

We constructed and verified a novel expression signature integrating of 5 MRGs, which was significantly associated with HCC prognosis, clinicopathological features, immune status, chemotherapeutic efficacy, and immunosuppressive molecules. The molecular mechanisms between MRGs and tumor activity in HCC are largely unknown and require further experimental investigation.

## Figures and Tables

**Table 1:** Baseline characteristics of HCC patients involved in this research.

Characteristics	TCGA-LIHC dataset (*N* = 365)	ICGC-LINC-JP dataset (*N* = 231)
Age		
≤60	173 (47.4%)	49 (21.2%)
>60	192 (52.6%)	182 (78.8%)

Gender		
Male	246 (67.4%)	170 (73.6%)
Female	119 (32.6%)	61 (26.4%)

Grade		
G1	55 (15.1%)	NA
G2	175 (47.9%)	NA
G3	118 (32.3%)	NA
G4	12 (3.3%)	NA
Unknown	5 (1.4%)	NA

Stage		
Stage I	170 (46.6%)	36 (15.6%)
Stage II	84 (23.0%)	105 (45.5%)
Stage III	83 (22.7%)	71 (30.7%)
Stage IV	4 (1.1%)	19 (8.2%)
Unknown	24 (6.6%)	0 (0.0%)

T stage		
T1-2	271 (74.2%)	NA
T3-4	91 (24.9%)	NA
Unknown	3 (0.8%)	NA

N stage		
N0	248 (67.9%)	NA
N1	4 (1.1%)	NA
Unknown	113 (31.0%)	NA

M stage		
M0	263 (72.1%)	NA
M1	3 (0.8%)	NA
Unknown	99 (27.1%)	NA

Survival status		
Alive	235 (64.4%)	189 (81.8%)
Deceased	130 (35.6%)	42 (18.2%)
Chronic liver disease/cirrhosis	NA	NA

HCC, hepatocellular carcinoma; TCGA, The Cancer Genome Atlas; LIHC, liver hepatocellular carcinoma; ICGC, International Cancer Genome Consortium.

## Data Availability

The expression and clinical data for HCC were downloaded from the TCGA-LIHC project (https://portal.gdc.cancer.gov/repository) and ICGC database (https://dcc.icgc.org/releases/current/Projects/LINC-JP).
